# Neutrophils: Orchestrators of the Malignant Phenotype

**DOI:** 10.3389/fimmu.2020.01778

**Published:** 2020-08-11

**Authors:** Brian E. Hsu, Yunyun Shen, Peter M. Siegel

**Affiliations:** ^1^Department of Medical Genetics, University of British Columbia, Vancouver, BC, Canada; ^2^Goodman Cancer Research Centre, McGill University, Montreal, QC, Canada; ^3^Department of Biochemistry, McGill University, Montreal, QC, Canada; ^4^Department of Medicine, McGill University, Montreal, QC, Canada

**Keywords:** neutrophils, tumor growth, metastasis, NETosis, immunosuppression, immunometabolism, metabolic plasticity/flexibility

## Abstract

Neutrophils are the first leukocytes recruited to sites of inflammation, where they execute anti-microbial functions to eliminate infectious agents. These functions include phagocytosis, release of reactive oxygen species and the formation of neutrophil extracellular traps via NETosis. Neutrophils are receiving increasing attention in the context of cancer, where these same neutrophil-associated functions are also important for modulating tumor growth and metastatic progression. Neutrophils are phenotypically heterogeneous and, depending on the context, exert anti- or pro-tumorigenic functions. Increasing evidence also suggests an important role of neutrophils and their involvement in promoting multiple steps of the metastatic cascade. The steps include: (1) local invasion and intravasation of cancer cells into circulation, (2) survival of cancer cells in the bloodstream and extravasation at a distant site, (3) early cancer cell seeding/survival, and (4) progressive growth of cancer cells to form macroscopic metastases. Although neutrophil functions designed to eliminate infectious agents can also eliminate tumor cells, their dysregulation can promote tumor growth and enable metastasis at multiple steps along the metastatic cascade. In this review, we will provide an overview of the current advances in neutrophil biology in the context of cancer. We also discuss the emerging field of immunometabolism, in which the rewiring of alternative metabolic pathways within neutrophils can impact their pro-tumorigenic/pro-metastatic functions.

## Introduction

Neutrophils account for 50–70% of circulating leukocytes and are the first immune cells recruited to an inflammatory site. They play an important role in the innate immune response to pathogens, as patients with neutropenia are highly susceptible to bacterial and fungal infections (1). Neutrophils perform numerous functions that target microbes, including phagocytosis, the release of anti-microbial peptides/proteases and NETosis (2). Interestingly, neutrophils have garnered considerable interest for their emerging and prominent roles in modulating cancer growth and metastatic progression (3). The roles played by neutrophils in the cancer setting are diverse and complex, leading to the concept of neutrophil heterogeneity/plasticity and the notion that distinct neutrophil subsets might exist.

Granulopoiesis is a tightly regulated process that involves the differentiation and mobilization of mature segmented neutrophils from the bone marrow into circulation. This process begins with the commitment of granulocyte-monocyte myeloid progenitors (GMPs), which progress through a series of neutrophil progenitors (myeloblast, pro-myelocyte, myelocyte, meta-myelocyte, band cell) until they become a mature neutrophil (4).

In cancer, dysregulated granulopoiesis has led to the identification of different neutrophil subsets that play a role in tumor progression. PreNeus comprise a neutrophil precursor population that retain their proliferative capacity and expand in the bone marrow and spleen of tumor bearing mice (5). PreNeus differentiate into immature and mature neutrophils, with the former found to accumulate in growing tumors (5). An early stage committed unipotent neutrophil precursor (NeP) has also been identified and their adoptive transfer into humanized mice promoted solid tumor growth by inhibiting T cell activation (6). Two neutrophil subsets, high-density neutrophils (HDNs), and low-density neutrophils (LDNs) were identified in various tumor models by differential density centrifugation (7). HDNs represent mature, segmented neutrophils whereas LDNs comprise a heterogeneous mixture of mature and immature neutrophils (7). Increasing mobilization of LDNs into the peripheral blood was associated with enhanced tumor growth and metastasis (7–9).

In addition to the identification of distinct neutrophil subsets, neutrophils exhibit plasticity in response to tumor-derived factors in a manner similar to macrophages. Neutrophils have been classified into two categories, N1 and N2, to describe their pro- and anti-tumorigenic functions, respectively, (10, 11). *In vivo* evidence has shown that tumor-associated neutrophils (TANs) can change their function from a pro-tumor phenotype (N2) to an anti-tumor (N1) phenotype with the addition of a TGFβ inhibitor, arguing that TGFβ is an important factor driving the N2 phenotype (10). In contrast, signals associated with an anti-tumor (N1) phenotype include type I interferons and those propagated by the MET receptor (12, 13). However, this categorization is likely to represent an oversimplification of neutrophil diversity. Neutrophil polarization, similar to macrophages, could also represent a continuum of different neutrophil phenotypes present in the tumor microenvironment (14). These advances regarding the degree of neutrophil heterogeneity/plasticity observed in the cancer setting have sparked an intense and renewed interest in this cell population. While there are ongoing discussions in the field regarding the relationship between PMN-MDSCs and neutrophil subsets, we direct the reader to excellent reviews that fully discuss these relationships (3, 15). We will briefly discuss anti-tumor neutrophil functions; however, this review will primarily discuss the recent roles of neutrophils and neutrophil-associated functions in promoting tumor growth and metastatic progression.

## Anti-Tumor Neutrophil Functions

Neutrophils can participate in a variety of anti-tumor mechanisms that limit tumor growth or eliminate cancer cells (16). A well-studied neutrophil-associated function is their ability to generate reactive oxygen species (ROS) to limit tumor progression. Upon tumor cell contact, mouse-derived neutrophils can release hydrogen peroxide to eliminate metastatic cancer cells *in vitro* (17). Subsequently, it was demonstrated that expression of TRPM2 (transient receptor potential cation channel, subfamily M2) on tumor cells increased their sensitivity to neutrophil-mediated, H_2_O_2_-dependent, cytotoxicity. This occurred through a mechanism that involved a transient increase in Ca^2+^ mobilization within cancer cells (18). TRPM2 upregulation in tumor cells occurred following an epithelial-to-mesenchymal transition (EMT) and cancer cells that have undergone an EMT were more susceptible to neutrophil-mediated killing (19). More recently, an interaction between the receptor for advanced glycation end products (RAGE), which is expressed on tumor cells, and cathepsin G present on murine neutrophils was shown to mediate *in vitro* tumor cell cytotoxicity in a H_2_O_2_-dependent manner (20). The release of neutrophil ROS is also dependent on the tumor microenvironment. In hypoxic tumor microenvironments, the ability of murine neutrophils to kill tumor cells *in vivo* through the release of ROS is greatly diminished (21). Thus, neutrophils have the capacity to mediate ROS-dependent direct tumor cell killing.

The interplay of neutrophils with other immune cell types can also indirectly limit tumor progression. Tumor associated neutrophils suppress the pro-tumorigenic role of IL-17 secreting γδ T cells by inhibiting their proliferation. Low glutathione levels in γδ17 T cells rendered them sensitive to neutrophil-derived ROS, causing enhanced oxidative stress, and reduced proliferation (22). In early-stage human lung cancer, a subset of immature neutrophils have been identified as having antigen-presenting functions and act to promote anti-tumor immunity by stimulating the secretion of inflammatory cytokines from T lymphocytes (23). In addition to neutrophil-T cell interactions, communication between neutrophils and monocytes can also elicit anti-tumor effects. Non-metastatic cancer cells can mobilize IFNγ-producing monocytes to the lungs. IFNγ release activates TMEM173/STING within neutrophils, which stimulates neutrophil-mediated killing of disseminated cancer cells in the lungs (24).

Neutrophils have been shown to infiltrate deposits of prostate cancer cells within bone metastases. Importantly, neutrophils impaired bone metastasis progression by inhibiting STAT5 (signal transducer and activator of transcription 5) function within prostate cancer cells, resulting in their apoptotic cell death (25). Recently, neutrophils have been reported to be involved in antibody-mediated trogocytosis, a process that mechanically disrupts the plasma membrane of antibody-opsinized cancer cells, leading to a lytic/necrotic-type cell death. IgA antibodies against receptors expressed by cancer cells (Her2, EGFR) could enhance neutrophil-mediated trogocytosis of cancer cells if the CD47-SIRPα innate immune cell checkpoint was simultaneously blocked (26, 27). Taken together, these results demonstrate that neutrophils can impair tumor growth and metastasis using a combination of direct and indirect cancer cell killing mechanisms (Supplementary Table 1).

## Neutrophil Functions That Promote Primary Tumor Growth

**Figure 1:**
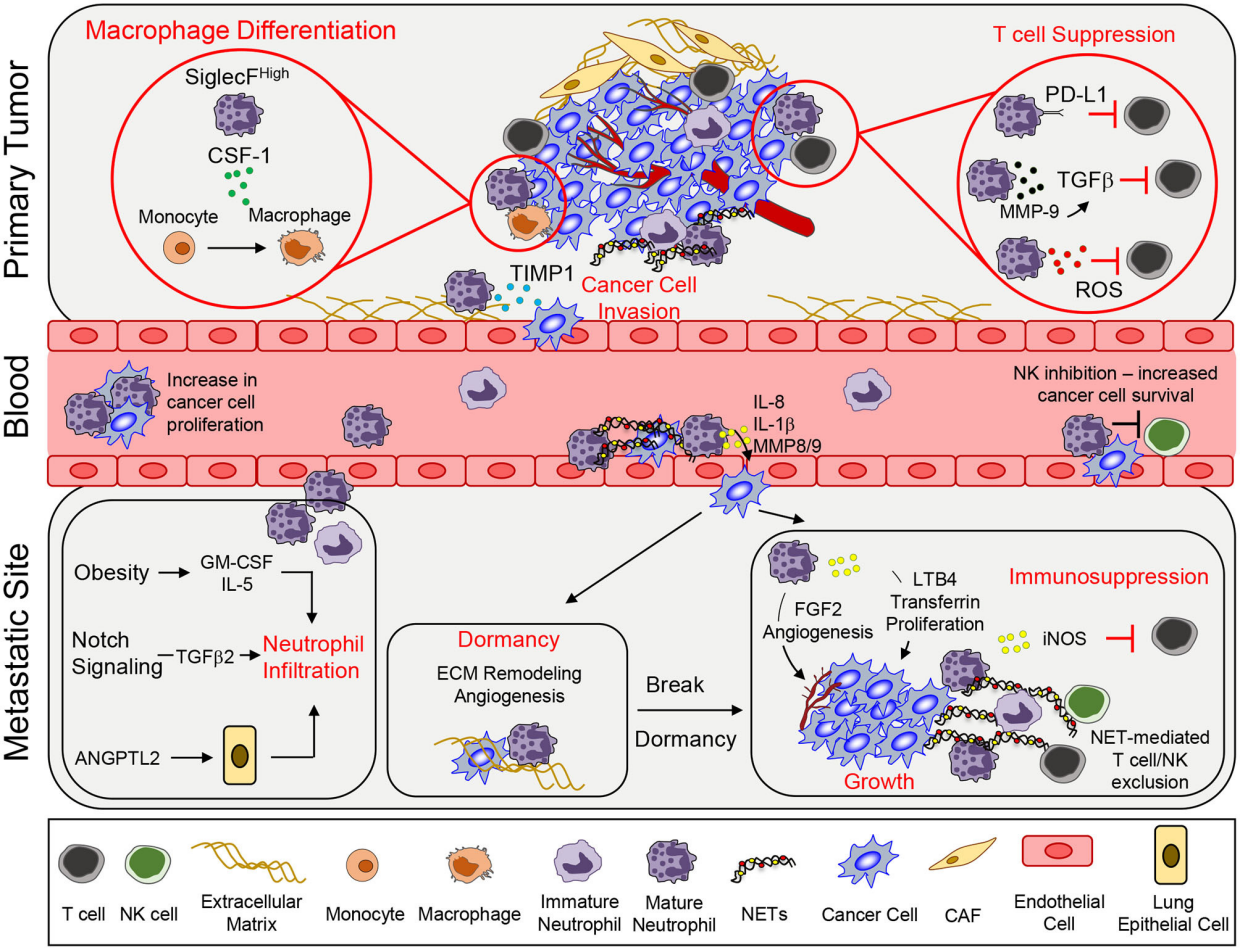
Neutrophil functions that promote tumor growth and metastasis. To support primary tumor growth, neutrophils can mediate T cell suppression and alter macrophage differentiation. Neutrophil release of TIMP-1 enhances tumor cell invasion by inducing epithelial-to-mesenchymal transition. Once in circulation, circulating tumor cells interact with neutrophils, which enables tumor cell proliferation. Secretion of various pro-inflammatory markers such as IL-8, IL-1β, or MMPs can mediate increased tumor cell extravasation. In addition, neutrophils can inhibit intraluminal NK-mediated killing of circulating cancer cells, leading to increased extravasation. At the metastatic site, various systemic, and microenvironmental factors can promote neutrophil infiltration. Neutrophils can awaken dormant cancer cells by promoting ECM remodeling and angiogenesis. Lastly, continued growth of the metastatic lesion is facilitated by key neutrophil-dependent mechanisms, which include angiogenesis, proliferation, immune suppression, and immune exclusion. CSF-1, colony stimulating factor 1; TIMP1, tissue inhibitor of matrix metalloprotease; PD-L1, programmed death ligand 1; TGFβ, transforming growth factor β; ROS, reactive oxygen species; MMP, matrix metalloproteinases; GM-CSF, granulocyte macrophage colony stimulating factor; ANGPTL2, angiopoetin like-2; FGF2, fibroblast growth factor 2; LTB_4_, leukotriene B_4_; iNOS, inducible nitric oxide synthase; NET, neutrophil extracellular trap; CAF, cancer-associated fibroblast.

Neutrophils promote primary tumor growth by various mechanisms (Figure 1). NETosis is a process that involves the extrusion of neutrophil-derived chromatin structures that are decorated with neutrophil granule constituents, which form extracellular structures called neutrophil extracellular traps (NETs) (28). Normally, NETosis and NET production have been described in the context of a neutrophil's ability to capture and kill bacteria extracellularly (29). However, NETs have been shown to play an important role in the growth of a primary tumor. Tumor microenvironmental changes, including tumor-associated coagulation and enhanced thrombosis, have been linked to enhanced tumor growth. Several recent studies suggest that NETosis may play an important role in these processes. LPS stimulation was shown to increase C3aR expression within neutrophils, enhance NETosis and increase coagulation. These events were correlated with N2 neutrophil polarization and increased tumor growth (30). Interestingly, it has recently been shown that immature neutrophils preferentially respond to cancer cell derived C3a to promote their migration (31). Subsequently, it was shown that breast cancer cells that expressed high levels of G-CSF and IL-1β exhibited high neutrophil counts and tumor-associated thrombosis, which was dependent on NET formation (32). Pharmacological blockade of IL-1 receptor signaling reduced NET formation, attenuated tumor-associated thrombosis and impaired tumor growth (32). NETs can also directly influence cancer cell proliferation. Neutrophil elastase (NE) present within NETs activates tumor cells to increase mitochondria biogenesis and ATP production; thereby, further enhancing the growth of cancer cells (33).

In addition to the impact of NETs, neutrophils can also interact with other immune cells through additional mechanisms to promote tumor growth. Neutrophil-derived ROS can inhibit T cell proliferation, creating an immunosuppressive environment that is supportive of tumor growth (34). Phenotypic characterization and single-cell RNA sequencing identified a neutrophil subset that is CD84^hi^, which exhibited potent T cell suppressive activity and increased ROS production (35). In a model of gastric cancer, neutrophils were activated by tumor-derived GM-CSF that resulted in elevated programmed death ligand 1 (PD-L1) expression. These PD-L1^+^ neutrophils were able to suppress T cell function and promote tumor growth (36). Secretion of MMP9 (matrix metalloproteinase 9) from infiltrating neutrophils activates latent TGF-β and induces T cell suppression and tumor growth in a colorectal cancer model (37). SiglecF^high^ neutrophils in lung adenocarcinoma created an immunosuppressive environment by promoting macrophage differentiation, causing the release of high levels of ROS and enabling tumor progression (38). Together, these findings indicate that neutrophils that infiltrate diverse primary tumors can modify the local environment in different ways to favor tumor growth.

## Neutrophil Functions That Promote Metastasis

The ability of cancer cells to leave the primary tumor and disseminate to distant organs represents the deadliest aspect of cancer progression. Indeed, the emergence of metastatic cancer accounts for ~90% of cancer related deaths (39). The metastatic cascade represents a series of barriers to cancer cells and neutrophils have been found to assist cancer cells in successfully navigating several of these distinct steps (Figure 1; Supplementary Table 1).

### Local Invasion/Intravasation

Infiltrating neutrophils within primary tumors are associated with an increase in EMT, enhanced metastasis and poor outcomes. Mechanistically, tissue inhibitor of matrix metalloprotease (TIMP-1) secreted by neutrophils induced an EMT and consequently increased the migration and invasion of tumor cells. Cancer cells that had undergone an EMT expressed CD90, which enhanced TIMP-1 secretion by neutrophils in a contact-dependent manner (40).

### Survival in Circulation/Extravasation

The ability of circulating tumor cells (CTCs) to survive is critical for metastasis formation (41). The formation of heterotypic cancer cell—neutrophil clusters was found to greatly increase metastatic fitness. Using a 4T1 breast cancer model, it was demonstrated that CTC-neutrophil interactions relied on VCAM-1 dependent adhesion, which enhanced cancer cell proliferation and increased metastasis (42). Indirectly, neutrophils can also inhibit NK cell-mediated tumor clearance in circulation; thereby increasing the intraluminal survival of disseminated tumor cells. In this study, 4T1 breast cancer cells were injected subcutaneously to mobilize murine neutrophils (Ly6G^+^), following which D2A1 breast cancer cells were injected intravenously. Mice bearing 4T1 cells exhibited reduced clearance of D2A1 cells from the lungs when compared to mice that were not injected with 4T1 cells (43). Depletion of NK cells resulted in enhanced D2A1 cancer cell accumulation in the lungs while neutrophil depletion had the opposite effect (43).

Cancer cells that have survived in circulation must exit the bloodstream and extravasate into tissue parenchyma (41). Neutrophils have been shown to regulate the extravasation process through several mechanisms. Neutrophil-derived factors can diminish the integrity of the endothelial barrier, permitting cancer cells to extravasate more easily. IL-8, IL-1β, and matrix metalloproteases (MMP8 and MMP9) released from neutrophils activated endothelial cells, reduced endothelial barrier function, increased transendothelial migration and accelerated the rate of cancer cell extravasation (43, 44).

NETosis, and NET constituents, can support cancer cell extravasation through enhanced trapping of CTCs within metastatic sites (45–48). Importantly, blocking NETosis decreases cancer cell adhesion and inhibits metastatic spread to the lung and liver (49, 50). Furthermore, changes within specific metastatic microenvironments, such as exposure to ozone or redox imbalance, triggered NETosis and led to increased entrapment of cancer cells in the lung and enhanced metastasis (51, 52). Collectively, these studies show that neutrophils play an important role in enhancing tumor cell survival and increased extravasation, which promote cancer metastasis.

### Early Seeding/Survival

Systemic and tumor-derived factors have been implicated in neutrophil recruitment in the pre-metastatic niche. Tumor-derived IL-1β induces γδ T cells to produce IL-17A and granulocyte-colony stimulating factor (G-CSF), which results in the recruitment of immunosuppressive neutrophils to the lung (8). GM-CSF and IL-5 have been shown to promote the expansion and recruitment of pro-metastatic neutrophils in the lungs of obese mice, which promotes lung metastasis (53). Angiopoetin-like-2 (ANGPTL2), secreted by osteosarcoma cells implanted in the tibia, stimulates lung epithelial cells, which led to the accumulation of neutrophils in the lung, and enhanced lung metastatic burden (54). In the lung, neutrophils secrete LTB_4_ that increases the proliferation of LTB_4_R-positive metastasis initiating cells (55). Activation of NOTCH1 in colorectal cancer cells drives TGFβ2-dependent recruitment of immunosuppressive neutrophils within the liver, which enabled the formation of liver metastases (56).

NETs also support early cancer cell seeding and colonization of metastases. Induction of NETs by ovarian tumor-derived factors has been shown to be important in promoting metastasis to the omentum (57). In the liver, NETs have also been shown to promote metastasis by activating cancer-associated fibroblasts (58).

### Growth in the Metastatic Site

Neutrophils have been shown to promote the growth of metastases after seeding. Minor subclones of breast cancer cells that secrete IL-11 and FIGF (C-fos-induced growth factor) can support the formation of polyclonal metastases composed of driver and passenger sub-populations. These IL-11 producing sub-clones activated IL-11 responsive mesenchymal stromal cells, which induced chemokine secretion and subsequent recruitment of pro-metastatic neutrophils (59). Tumor cell-derived GM-CSF was shown to stimulate neutrophils to synthesize and secrete transferrin, an iron transport protein, which has mitogenic activity that promotes lung metastatic growth when taken up by cancer cells (60).

A recurring function of pro-metastatic neutrophils is their ability to create an immunosuppressive microenvironment that support metastasis. Within lung metastases, inducible nitric oxide synthase (iNOS) producing neutrophils have been shown to limit CD8^+^ T cell dependent anti-tumor responses by promoting immune suppression (8). Recently, p53-deficient cancer cells were found to increase the expression of Wnt ligands, which in turn upregulated IL-1β production from tumor-associated macrophages (61) High IL-1β levels engaged γδ17 T cells, which subsequently enhanced neutrophil recruitment that promoted the formation of lung metastases (61). Furthermore, loss of Elf5 (E74-like transcription factor) expression in triple-negative breast cancer led to increased IFN-γ signaling resulting in the expansion of immunosuppressive neutrophils (62). In addition to tumor-derived factors, a lack of systemic testosterone levels can lead to an impairment of anti-tumor neutrophil functions. A shift toward immature neutrophils was observed in castrated male mice, leading to increased neutrophil-derived ROS and suppression of NK cell activation that promoted increased lung metastatic burden in two melanoma models (63). Recently, a role for NET formation has been described for the continued growth of established metastases (64). NETs released during cancer progression was shown to limit the ability of NK and cytotoxic T cells to eliminate cancer cells. Specifically, NET formation impaired direct contact between the cancer cells and cytotoxic immune cells (NK and T cells). Inhibition of NETosis with a protein arginine deiminase 4 (PAD4) inhibitor synergized with immune checkpoint inhibitors to control tumor growth and metastasis (64).

Pro-angiogenic functions have long been ascribed for neutrophils, which revealed that neutrophil-derived proteases (such as MMP9) could release stored angiogenic factors (VEGF, FGFs) that were stored in the local environment to enable blood vessel formation (65, 66). Recently, a different mechanism by which neutrophils enhance angiogenesis has been described. The synthesis and secretion of fibroblast growth factor 2 (FGF2) by neutrophils in the liver microenvironment drives angiogenesis and growth of nascent colorectal cancer-derived hepatic metastases (67).

## Dormant/Residual Disease and Therapy Resistance

Neutrophils have also been implicated in awakening dormant cancer cells. LPS-induced tissue inflammation led to metastatic outgrowth of dormant tumor cells in a neutrophil-dependent manner (68). MMP-9 produced by neutrophils can trigger the growth of dormant cancer cells by remodeling extracellular matrix and releasing potent angiogenic factors (69). NE and MMP-9, which are enzymes associated with NETs, can cleave the extracellular matrix (ECM) leading to integrin-mediated signaling, which awakens dormant cancer cells and promotes cancer cell growth (70).

Several studies have shown that neutrophils promote resistance to therapy. Doxorubicin and paclitaxel resistant breast cancer cells express more IL-17 and CXCR2 ligands, which increases neutrophil recruitment (71). A neutrophil-enriched subtype characterized in triple negative breast cancer (TNBC) determined that neutrophils were largely immunosuppressive, rendering these tumors resistant to immune checkpoint blockade therapy (72). In a genetically engineered mouse model of sarcoma, neutrophils promote resistance to radiation therapy by activating mitogen-activated protein kinase (MAPK) pathway (73). In addition, CD177^+^ neutrophil infiltrates in colorectal cancer patients are associated with adverse outcome in patients receiving bevacizumab [anti-vascular endothelial growth factor A (VEGF- A)] (74). Furthermore, Lysyl oxidase-like 4 (LOXL4) expressing neutrophils that infiltrated colorectal cancer liver metastases were found to identify patients that were resistant to anti-angiogenic therapy (75).

## Metabolic Programming in Neutrophils

**Figure 2:**
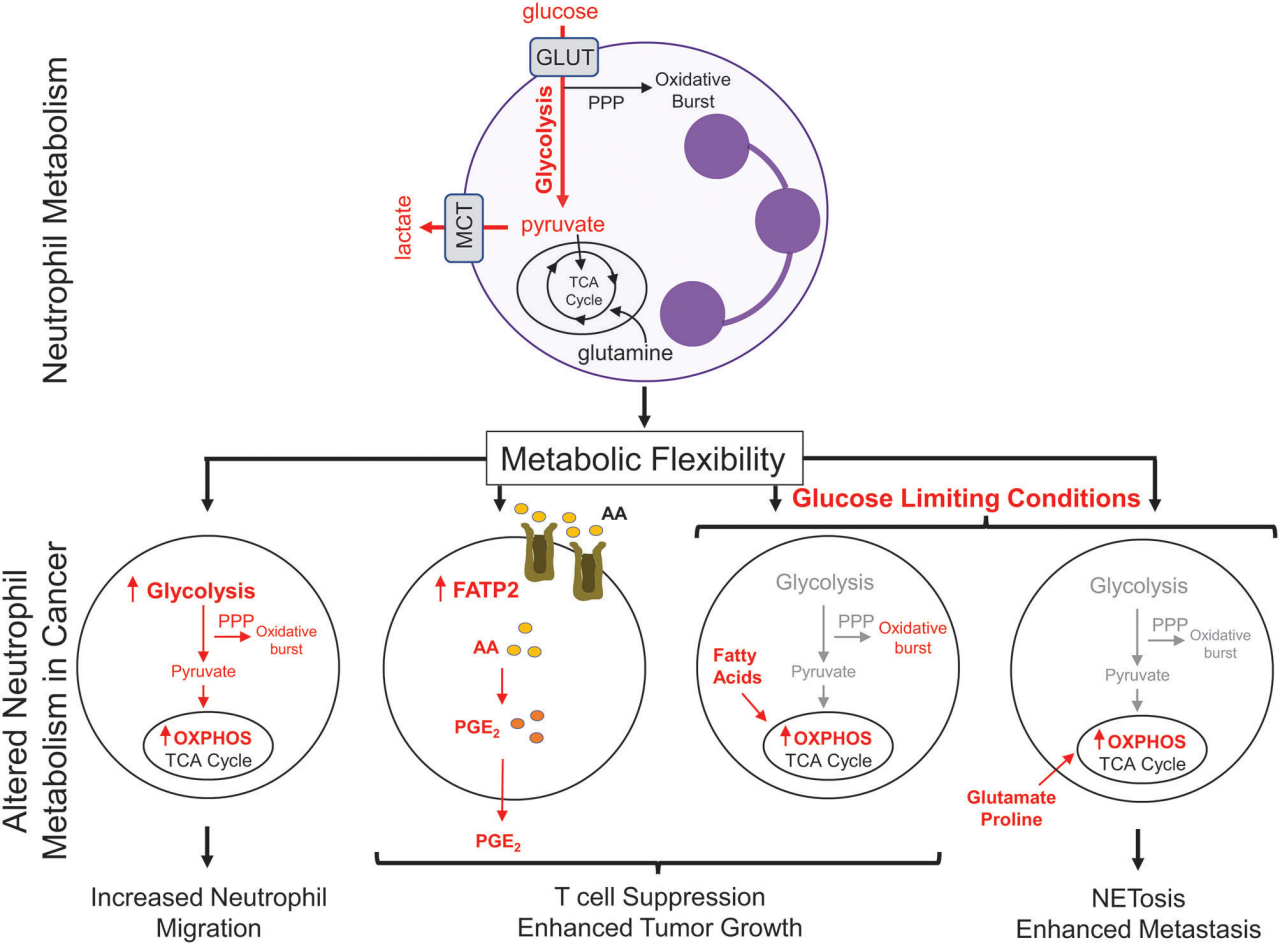
Metabolic changes in cancer-associated neutrophils. Neutrophils, which possess few mitochondria, are reliant on glycolysis to generate ATP to fuel effector functions such as phagocytosis, generation of reactive oxygen species, and NETosis. In cancer, neutrophils upregulate oxidative phosphorylation (OXPHOS) and fatty acid transporters to mediate many neutrophil functions; including migration and T cell suppression. Under nutrient limiting conditions, such as low glucose, neutrophils can reprogram their metabolism to break down fatty acids or utilize certain amino acids (glutamate, proline) to fuel pro-tumorigenic/pro-metastatic functions. PPP, pentose phosphate pathway; GLUT, glucose transporter; MCT, Monocarboxylate transporter 1; TCA, tricarboxylic acid cycle; FATP2, fatty acid transport protein 2; AA, arachidonic acid; PGE_2_, prostaglandin E2.

There has been recent interest in the concept of immunometabolism and the realization that altered cellular metabolism in infiltrating immune cells can have a significant impact on tumor growth and metastasis (76). Neutrophils are typically viewed as a cell type that is heavily reliant on glycolysis to perform their effector functions (77). Consistent with this notion, neutrophils have very few mitochondria and inhibitors of oxidative phosphorylation (OXPHOS) do not alter their rates of oxygen consumption (77, 78). However, during tumor progression, neutrophils have been shown to undergo a metabolic switch, which involves the upregulation of genes associated with OXPHOS, fatty acid metabolism, and glycolysis (Figure 2) (38). Neutrophils isolated from Lewis lung carcinoma exhibit increased flux through OXPHOS, glycolysis, and increased ATP production compared to naïve neutrophils, suggesting that multiple metabolic strategies are engaged in tumor infiltrating neutrophils (79). Recently, upregulation of FATP2 (fatty acid transport protein 2) in neutrophils was shown to increase lipid accumulation in these cells. FATP2 regulated the uptake of arachidonic acid, which was subsequently converted to prostaglandin E2. Neutrophil-derived prostaglandin E2 was found to be important or neutrophil-mediated CD8^+^ T cell suppression and tumor growth (80).

Metabolic flexibility refers to the ability of a cell to shift between one metabolic program to another in response to changing metabolic demands or nutrient supply. High metabolic flexibility increases the cell's ability to survive various and everchanging metabolic microenvironments (81). Neutrophil sub-populations can also exhibit metabolic flexibility (Figure 2). In breast cancer, splenic neutrophils can engage mitochondrial-dependent fatty acid oxidation as a predominate fuel source to support ROS production and maintain T cell suppression (82). Under glucose-limiting conditions, similar to certain tumor microenvironments, immature LDNs have been shown to utilize OXPHOS to generate ATP that is required to support their pro-tumorigenic functions. Indeed, immature LDNs can support NETosis under nutrient limiting conditions via mitochondrial-dependent amino acid catabolism, which is important for efficient breast cancer liver metastasis (9). In addition, the longevity of neutrophils could also be altered due to the enhanced metabolic flexibility. The *ex vivo* half-life of mouse circulating HDNs and LDNs was 4 and 12 h, respectively (7, 9). Such observations raise the intriguing possibility that, under certain conditions, distinct neutrophil subsets may not be as short-lived as previously thought. These studies argue that increased metabolic flexibility in distinct neutrophil populations may be important for cellular functions that can influence tumor growth and metastatic progression.

## Clinical Importance: Future Perspectives on Treatment

In keeping with their pro-tumorigenic/metastatic functions, the presence of neutrophils across 25 different cancers was shown to be strongly associated with adverse patient outcomes (83). Among certain subtypes of breast cancer (ER-), the presence of a neutrophil infiltrate in the primary tumor is also indicative of worse patient outcomes (84). Furthermore, in patients with advanced cancers, serum IL-8 levels, and neutrophil infiltration are associated with worse overall survival and diminished response to immune checkpoint inhibitors (85).

The mobilization of neutrophils into circulation also has prognostic significance. The neutrophil-to-lymphocyte ratio (NLR) is an important risk stratification and treatment selection diagnostic tool for cancer patients. A high NLR is associated with poor prognosis in many solid human cancers (86–96). A high NLR is also associated with decreased overall survival in patients with TNBC or metastatic breast cancer (97, 98).

An important and unanswered question with respect to the NLR is the type of neutrophil that is being detected in these patients, are they high- or low-density neutrophils? Interestingly, LDNs have been identified in patients with breast cancer, lung cancer, head and neck cancers, urologic cancers, and lymphoma (7, 99–101). In patients with advanced lung cancer, it was reported that higher proportion of LDNs (>10%) predicted poorer survival (102). These observations are in keeping with the pro-tumorigenic and pro-metastatic functions associated with LDN/N2 neutrophils. While most studies reveal a negative prognostic impact of neutrophils in cancer, there was one study that associated the presence of a CD16^high^ CD62^dim^ neutrophil subset with increased survival of head and neck squamous cell carcinoma patients (103). These observations highlight the need for better markers that are capable of discriminating between neutrophils that exert anti-tumor vs. those that mediate pro-tumor/metastatic effects.

Mechanistic insights have greatly advanced our knowledge of tumor-derived factors that impact tumor growth and metastasis in a neutrophil-dependent manner. Additional studies focused on characterizing the phenotypic and functional role of neutrophils in cancer, it may be possible to develop strategies that specifically target those neutrophil subsets that actively promote tumor growth and metastasis, while sparing those neutrophils that possess anti-tumor and anti-microbial functions. Finally, the emerging concept of metabolic flexibility that is exhibited by certain neutrophil subsets may afford new ways of targeting these pro-tumorigenic/metastatic neutrophils.

## Author Contributions

BH, YS, and PS wrote the review and prepared the figures. All authors contributed to the article and approved the submitted version.

## Conflict of Interest

The authors declare that the research was conducted in the absence of any commercial or financial relationships that could be construed as a potential conflict of interest.

## References

[1] Sipsas NV Bodey GP Kontoyiannis DP. Perspectives for the management of febrile neutropenic patients with cancer in the 21st century. Cancer. (2005) 103:1103–13. 10.1002/cncr.20890 15666328

[2] Kolaczkowska E Kubes P. Neutrophil recruitment and function in health and inflammation. Nat Rev Immunol. (2013) 13:159–75. 10.1038/nri3399 23435331

[3] Coffelt SB Wellenstein MD de Visser KE. Neutrophils in cancer: neutral no more. Nat Rev Cancer. (2016) 16:431–46. 10.1038/nrc.2016.52 27282249

[4] Cowland JB Borregaard N. Granulopoiesis and granules of human neutrophils. Immunol Rev. (2016) 273:11–28. 10.1111/imr.12440 27558325

[5] Evrard M Kwok IWH Chong SZ Teng KWW Becht E Chen J. Developmental analysis of bone marrow neutrophils reveals populations specialized in expansion, trafficking, and effector functions. Immunity. (2018) 48:364–79.e8. 10.1016/j.immuni.2018.02.002 29466759

[6] Zhu YP Padgett L Dinh HQ Marcovecchio P Blatchley A Wu R. Identification of an early unipotent neutrophil progenitor with pro- tumoral activity in mouse and human bone marrow. Cell Rep. (2018) 24:2329–41.e8. 10.1016/j.celrep.2018.07.097 30157427 PMC6542273

[7] Sagiv JY Michaeli J Assi S Mishalian I Kisos H Levy L. Phenotypic diversity and plasticity in circulating neutrophil subpopulations in cancer. Cell Rep. (2015) 10:562–73. 10.1016/j.celrep.2014.12.039 25620698

[8] Coffelt SB Kersten K Doornebal CW Weiden J Vrijland K Hau CS. IL-17-producing gammadelta T cells and neutrophils conspire to promote breast cancer metastasis. Nature. (2015) 522:345–8. 10.1038/nature14282 25822788 PMC4475637

[9] Hsu BE Tabaries S Johnson RM Andrzejewski S Senecal J Lehuede C. Immature low-density neutrophils exhibit metabolic flexibility that facilitates breast cancer liver metastasis. Cell Rep. (2019) 27:3902–15.e6. 10.1016/j.celrep.2019.05.091 31242422

[10] Fridlender ZG Sun J Kim S Kapoor V Cheng G Ling L. Polarization of tumor-associated neutrophil phenotype by TGF-beta: “N1” versus “N2” TAN. Cancer Cell. (2009) 16:183–94. 10.1016/j.ccr.2009.06.017 19732719 PMC2754404

[11] Ohms M Möller S Laskay T. An attempt to polarize human neutrophils toward N1 and N2 phenotypes *in vitro*. Front Immunol. (2020) 11:532. 10.3389/fimmu.2020.00532 32411122 PMC7198726

[12] Jablonska J Leschner S Westphal K Lienenklaus S Weiss S. Neutrophils responsive to endogenous IFN-beta regulate tumor angiogenesis and growth in a mouse tumor model. J Clin Invest. (2010) 120:1151–64. 10.1172/JCI37223 20237412 PMC2846036

[13] Finisguerra V Di Conza G Di Matteo M Serneels J Costa S Thompson AA. MET is required for the recruitment of anti-tumoural neutrophils. Nature. (2015) 522:349–53. 10.1038/nature14407 25985180 PMC4594765

[14] Ostuni R Kratochvill F Murray PJ Natoli G. Macrophages and cancer: from mechanisms to therapeutic implications. Trends Immunol. (2015) 36:229–39. 10.1016/j.it.2015.02.004 25770924

[15] Brandau S Moses K Lang S The kinship of neutrophils and granulocytic myeloid-derived suppressor cells in cancer: cousins, siblings or twins? Semin Cancer Biol. (2013) 23:171–82. 10.1016/j.semcancer.2013.02.007 23459190

[16] Vols S Sionov RV Granot Z. Always look on the bright side: anti-tumor functions of neutrophils. Curr Pharmac Design. (2017) 23:4862–92. 10.2174/1381612823666170704125420 28677504

[17] Granot Z Henke E Comen EA King TA Norton L Benezra R. Tumor entrained neutrophils inhibit seeding in the premetastatic lung. Cancer Cell. (2011) 20:300–14. 10.1016/j.ccr.2011.08.012 21907922 PMC3172582

[18] Gershkovitz M Caspi Y Fainsod-Levi T Katz B Michaeli J Khawaled S. TRPM2 mediates neutrophil killing of disseminated tumor cells. Cancer Res. (2018) 78:2680–90. 10.1158/0008-5472.CAN-17-3614 29490946

[19] Gershkovitz M Fainsod-Levi T Khawaled S Shaul ME Sionov RV Cohen-Daniel L. Microenvironmental cues determine tumor cell susceptibility to neutrophil cytotoxicity. Cancer Res. (2018) 78:5050–9. 10.1158/0008-5472.CAN-18-0540 29967257

[20] Sionov RV Fainsod-Levi T Zelter T Polyansky L Pham CT Granot Z. Neutrophil cathepsin G and tumor cell rage facilitate neutrophil anti- tumor cytotoxicity. Oncoimmunology. (2019) 8:e1624129. 10.1080/2162402X.2019.1624129 31428521 PMC6685517

[21] Mahiddine K Blaisdell A Ma S Crequer-Grandhomme A Lowell CA Erlebacher A. Relief of tumor hypoxia unleashes the tumoricidal potential of neutrophils. J Clin Invest. (2020) 130:389–403. 10.1172/JCI130952 31600172 PMC6934192

[22] Mensurado S Rei M Lanca T Ioannou M Goncalves-Sousa N Kubo H. Tumor-associated neutrophils suppress pro-tumoral IL-17+ gammadelta T cells through induction of oxidative stress. PLoS Biol. (2018) 16:e2004990. 10.1371/journal.pbio.2004990 29750788 PMC5965901

[23] Singhal S Bhojnagarwala PS O'Brien S Moon EK Garfall AL Rao AS. Origin and role of a subset of tumor-associated neutrophils with antigen- presenting cell features in early-stage human lung cancer. Cancer Cell. (2016) 30:120–35. 10.1016/j.ccell.2016.06.001 27374224 PMC4945447

[24] Hagerling C Gonzalez H Salari K Wang CY Lin C Robles I. Immune effector monocyte-neutrophil cooperation induced by the primary tumor prevents metastatic progression of breast cancer. Proc Natl Acad Sci USA. (2019) 116:21704–14. 10.1073/pnas.1907660116 31591235 PMC6815161

[25] Costanzo-Garvey DL Keeley T Case AJ Watson GF Alsamraae M Yu Y. Neutrophils are mediators of metastatic prostate cancer progression in bone. Cancer Immunol Immunother. (2020) 69:1113–30. 10.1007/s00262-020-02527-6 32114681 PMC7230043

[26] Matlung HL Babes L Zhao XW van Houdt M Treffers LW van Rees DJ. Neutrophils kill antibody-opsonized cancer cells by trogoptosis. Cell Rep. (2018) 23:3946–59.e6. 10.1016/j.celrep.2018.05.082 29949776

[27] Treffers LW Ten Broeke T Rosner T Jansen JHM van Houdt M Kahle S. IgA-mediated killing of tumor cells by neutrophils is enhanced by CD47-SIRPα checkpoint inhibition. Cancer Immunol Res. (2020) 8:120–30. 10.1158/2326-6066.CIR-19-0144 31690649

[28] Papayannopoulos V. Neutrophil extracellular traps in immunity and disease. Nat Rev Immunol. (2018) 18:134–47. 10.1038/nri.2017.105 28990587

[29] Brinkmann V Reichard U Goosmann C Fauler B Uhlemann Y Weiss DS. Neutrophil extracellular traps kill bacteria. Science. (2004) 303:1532–5. 10.1126/science.1092385 15001782

[30] Guglietta S Chiavelli A Zagato E Krieg C Gandini S Ravenda PS. Coagulation induced by C3aR-dependent NETosis drives protumorigenic neutrophils during small intestinal tumorigenesis. Nat Commun. (2016) 7:11037. 10.1038/ncomms11037 26996437 PMC4802169

[31] Hsu BE Roy J Mouhanna J Rayes RF Ramsay L Tabaries S. C3a elicits unique migratory responses in immature low-density neutrophils. Oncogene. (2020) 39:2612–23. 10.1038/s41388-020-1169-8 32020055

[32] Gomes T Varady CBS Lourenco AL Mizurini DM Rondon AMR Leal AC. IL-1beta blockade attenuates thrombosis in a neutrophil extracellular trap-dependent breast cancer model. Front Immunol. (2019) 10:2088. 10.3389/fimmu.2019.02088 31552036 PMC6737452

[33] Yazdani HO Roy E Comerci AJ van der Windt DJ Zhang H Huang H. Neutrophil extracellular traps drive mitochondrial homeostasis in tumors to augment growth. Cancer Res. (2019) 79:5626–39. 10.1158/0008-5472.CAN-19-0800 31519688 PMC6825588

[34] Casbon AJ Reynaud D Park C Khuc E Gan DD Schepers K. Invasive breast cancer reprograms early myeloid differentiation in the bone marrow to generate immunosuppressive neutrophils. Proc Natl Acad Sci USA. (2015) 112:E566–75. 10.1073/pnas.1424927112 25624500 PMC4330753

[35] Alshetaiwi H Pervolarakis N McIntyre LL Ma D Nguyen Q Rath JA. Defining the emergence of myeloid-derived suppressor cells in breast cancer using single-cell transcriptomics. Sci Immunol. (2020) 5:eaay6017. 10.1126/sciimmunol.aay6017 32086381 PMC7219211

[36] Wang TT Zhao YL Peng LS Chen N Chen W Lv YP. Tumour-activated neutrophils in gastric cancer foster immune suppression and disease progression through GM-CSF-PD-L1 pathway. Gut. (2017) 66:1900–11. 10.1136/gutjnl-2016-313075 28274999 PMC5739867

[37] Germann M Zangger N Sauvain MO Sempoux C Bowler AD Wirapati P. Neutrophils suppress tumor-infiltrating T cells in colon cancer via matrix metalloproteinase-mediated activation of TGFβ. EMBO Mol Med. (2020) 12:e10681. 10.15252/emmm.201910681 31793740 PMC6949488

[38] Engblom C Pfirschke C Zilionis R Da Silva Martins J Bos SA Courties G. Osteoblasts remotely supply lung tumors with cancer-promoting SiglecF (high) neutrophils. Science. (2017) 358:eaal5081. 10.1126/science.aal5081 29191879 PMC6343476

[39] Chaffer CL Weinberg RA. A perspective on cancer cell metastasis. Science. (2011) 331:1559. 10.1126/science.1203543 21436443

[40] Wang Y Chen J Yang L Li J Wu W Huang M. Tumor-contacted neutrophils promote metastasis by a CD90-TIMP-1 juxtacrine-paracrine loop. Clin Cancer Res. (2019) 25:1957–69. 10.1158/1078-0432.CCR-18-2544 30482778

[41] Massagué J Obenauf AC. Metastatic colonization by circulating tumour cells. Nature. (2016) 529:298–306. 10.1038/nature17038 26791720 PMC5029466

[42] Szczerba BM Castro-Giner F Vetter M Krol I Gkountela S Landin J. Neutrophils escort circulating tumour cells to enable cell cycle progression. Nature. (2019) 566:553–7. 10.1038/s41586-019-0915-y 30728496

[43] Spiegel A Brooks MW Houshyar S Reinhardt F Ardolino M Fessler E. Neutrophils Suppress intraluminal NK cell-mediated tumor cell clearance and enhance extravasation of disseminated carcinoma cells. Cancer Discov. (2016) 6:630–49. 10.1158/2159-8290.CD-15-1157 27072748 PMC4918202

[44] Chen MB Hajal C Benjamin DC Yu C Azizgolshani H Hynes RO. Inflamed neutrophils sequestered at entrapped tumor cells via chemotactic confinement promote tumor cell extravasation. Proc Natl Acad Sci USA. (2018) 115:7022–7. 10.1073/pnas.1715932115 29915060 PMC6142213

[45] Park J Wysocki RW Amoozgar Z Maiorino L Fein MR Jorns J. Cancer cells induce metastasis-supporting neutrophil extracellular DNA traps. Sci Transl Med. (2016) 8:361ra138. 10.1126/scitranslmed.aag1711 27798263 PMC5550900

[46] Rayes RF Vourtzoumis P Bou Rjeily M Seth R Bourdeau F Giannias B. Neutrophil extracellular trap-associated CEACAM1 as a putative therapeutic target to prevent metastatic progression of colon carcinoma. J Immunol. (2020) 204:2285–94. 10.4049/jimmunol.1900240 32169849 PMC7534954

[47] Najmeh S Cools-Lartigue J Rayes RF Gowing S Vourtzoumis P Bourdeau F. Neutrophil extracellular traps sequester circulating tumor cells via beta1-integrin mediated interactions. Int J Cancer. (2017) 140:2321–30. 10.1002/ijc.30635 28177522

[48] Cools-Lartigue J Spicer J McDonald B Gowing S Chow S Giannias B. Neutrophil extracellular traps sequester circulating tumor cells and promote metastasis. J Clin Invest. (2013) 123:3446–58. 10.1172/JCI67484 23863628 PMC3726160

[49] Rayes RF Mouhanna JG Nicolau I Bourdeau F Giannias B Rousseau S. Primary tumors induce neutrophil extracellular traps with targetable metastasis promoting effects. JCI Insight. (2019) 5:e128008. 10.1158/1538-7445.AM2019-1508 31343990 PMC6777835

[50] Yang LY Luo Q Lu L Zhu WW Sun HT Wei R. Increased neutrophil extracellular traps promote metastasis potential of hepatocellular carcinoma via provoking tumorous inflammatory response. J Hematol Oncol. (2020) 13:3. 10.1186/s13045-019-0836-0 31907001 PMC6945602

[51] Rocks N Vanwinge C Radermecker C Blacher S Gilles C Maree R. Ozone-primed neutrophils promote early steps of tumour cell metastasis to lungs by enhancing their NET production. Thorax. (2019) 74:768–79. 10.1136/thoraxjnl-2018-211990 31142617

[52] Inoue M Nakashima R Enomoto M Koike Y Zhao X Yip K. Plasma redox imbalance caused by albumin oxidation promotes lung- predominant NETosis and pulmonary cancer metastasis. Nat Commun. (2018) 9:5116. 10.1038/s41467-018-07550-x 30504805 PMC6269536

[53] Quail DF Olson OC Bhardwaj P Walsh LA Akkari L Quick ML. Obesity alters the lung myeloid cell landscape to enhance breast cancer metastasis through IL5 and GM-CSF. Nat Cell Biol. (2017) 19:974–87. 10.1038/ncb3578 28737771 PMC6759922

[54] Charan M Dravid P Cam M Setty B Roberts RD Houghton PJ. Tumor secreted ANGPTL2 facilitates recruitment of neutrophils to the lung to promote lung pre-metastatic niche formation and targeting ANGPTL2 signaling affects metastatic disease. Oncotarget. (2020) 11:510–22. 10.18632/oncotarget.27433 32082485 PMC7007290

[55] Wculek SK Malanchi I. Neutrophils support lung colonization of metastasis-initiating breast cancer cells. Nature. (2015) 528:413–7. 10.1038/nature16140 26649828 PMC4700594

[56] Jackstadt R van Hooff SR Leach JD Cortes-Lavaud X Lohuis JO Ridgway RA. Epithelial NOTCH signaling rewires the tumor microenvironment of colorectal cancer to drive poor-prognosis subtypes and metastasis. Cancer Cell. (2019) 36:319–36.e7. 10.1016/j.ccell.2019.08.003 31526760 PMC6853173

[57] Lee W Ko SY Mohamed MS Kenny HA Lengyel E Naora H. Neutrophils facilitate ovarian cancer premetastatic niche formation in the omentum. J Exp Med. (2019) 216:176–94. 10.1084/jem.20181170 30567719 PMC6314534

[58] Takesue S Ohuchida K Shinkawa T Otsubo Y Matsumoto S Sagara A. Neutrophil extracellular traps promote liver micrometastasis in pancreatic ductal adenocarcinoma via the activation of cancerassociated fibroblasts. Int J Oncol. (2020) 56:596–605. 10.3892/ijo.2019.4951 31894273

[59] Janiszewska M Tabassum DP Castano Z Cristea S Yamamoto KN Kingston NL. Subclonal cooperation drives metastasis by modulating local and systemic immune microenvironments. Nat Cell Biol. (2019) 21:879–88. 10.1038/s41556-019-0346-x 31263265 PMC6609451

[60] Liang W Li Q Ferrara N. Metastatic growth instructed by neutrophil-derived transferrin. Proc Natl Acad Sci USA. (2018) 115:11060–5. 10.1073/pnas.1811717115 30301793 PMC6205468

[61] Wellenstein MD Coffelt SB Duits DEM van Miltenburg MH Slagter M de Rink I. Loss of p53 triggers WNT-dependent systemic inflammation to drive breast cancer metastasis. Nature. (2019) 572:538–42. 10.1038/s41586-019-1450-6 31367040 PMC6707815

[62] Singh S Kumar S Srivastava RK Nandi A Thacker G Murali H. Loss of ELF5–FBXW7 stabilizes IFNGR1 to promote the growth and metastasis of triple-negative breast cancer through interferon-γ signalling. Nat Cell Biol. (2020) 22:591–602. 10.1038/s41556-020-0495-y 32284542 PMC8237104

[63] Markman JL Porritt RA Wakita D Lane ME Martinon D Noval Rivas M. Loss of testosterone impairs anti-tumor neutrophil function. Nat Commun. (2020) 11:1613. 10.1038/s41467-020-15397-4 32235862 PMC7109066

[64] Teijeira Á Garasa S Gato M Alfaro C Migueliz I Cirella A. CXCR1 and CXCR2 chemokine receptor agonists produced by tumors induce neutrophil extracellular traps that interfere with immune cytotoxicity. Immunity. (2020) 52:856–71.e8. 10.1016/j.immuni.2020.03.001 32289253

[65] Ishai-Michaeli R Eldor A Vlodavsky I. Heparanase activity expressed by platelets, neutrophils, and lymphoma cells releases active fibroblast growth factor from extracellular matrix. Cell Regul. (1990) 1:833–42. 10.1091/mbc.1.11.833 2088528 PMC362850

[66] Nozawa H Chiu C Hanahan D. Infiltrating neutrophils mediate the initial angiogenic switch in a mouse model of multistage carcinogenesis. Proc Natl Acad Sci USA. (2006) 103:12493–8. 10.1073/pnas.0601807103 16891410 PMC1531646

[67] Gordon-Weeks AN Lim SY Yuzhalin AE Jones K Markelc B Kim KJ. Neutrophils promote hepatic metastasis growth through fibroblast growth factor 2-dependent angiogenesis in mice. Hepatology. (2017) 65:1920–35. 10.1002/hep.29088 28133764

[68] De Cock JM Shibue T Dongre A Keckesova Z Reinhardt F Weinberg RA. Inflammation triggers zeb1-dependent escape from tumor latency. Cancer Res. (2016) 76:6778–84. 10.1158/0008-5472.CAN-16-0608 27530323 PMC5135644

[69] Luo J Feng XX Luo C Wang Y Li D Shu Y. 14, 15-EET induces the infiltration and tumor-promoting function of neutrophils to trigger the growth of minimal dormant metastases. Oncotarget. (2016) 7:43324–36. 10.18632/oncotarget.9709 27270316 PMC5190026

[70] Albrengues J Shields MA Ng D Park CG Ambrico A Poindexter ME. Neutrophil extracellular traps produced during inflammation awaken dormant cancer cells in mice. Science. (2018) 361:eaao4227. 10.1126/science.aao4227 30262472 PMC6777850

[71] Wu L Awaji M Saxena S Varney ML Sharma B Singh RK. IL-17-CXC chemokine receptor 2 axis facilitates breast cancer progression by up-regulating neutrophil recruitment. Am J Pathol. (2020) 190:222–33. 10.1016/j.ajpath.2019.09.016 31654638 PMC6943375

[72] Kim IS Gao Y Welte T Wang H Liu J Janghorban M. Immuno-subtyping of breast cancer reveals distinct myeloid cell profiles and immunotherapy resistance mechanisms. Nat Cell Biol. (2019) 21:1113–26. 10.1038/s41556-019-0373-7 31451770 PMC6726554

[73] Wisdom AJ Hong CS Lin AJ Xiang Y Cooper DE Zhang J. Neutrophils promote tumor resistance to radiation therapy. Proc Natl Acad Sci USA. (2019) 116:18584–9. 10.1073/pnas.1901562116 31462499 PMC6744874

[74] Schiffmann LM Fritsch M Gebauer F Gunther SD Stair NR Seeger JM. Tumour-infiltrating neutrophils counteract anti-VEGF therapy in metastatic colorectal cancer. Br J Cancer. (2019) 120:69–78. 10.1038/s41416-018-0198-3 30377339 PMC6325148

[75] Palmieri V Lazaris A Mayer TZ Petrillo SK Alamri H Rada M. Neutrophils expressing lysyl oxidase-like 4 protein are present in colorectal cancer liver metastases resistant to anti-angiogenic therapy. J Pathol. (2020) 251:213–23. 10.1002/path.5449 32297656

[76] Buck MD Sowell RT Kaech SM Pearce EL. Metabolic instruction of immunity. Cell. (2017) 169:570–86. 10.1016/j.cell.2017.04.004 28475890 PMC5648021

[77] Kramer PA Ravi S Chacko B Johnson MS Darley-Usmar VM. A review of the mitochondrial and glycolytic metabolism in human platelets and leukocytes: implications for their use as bioenergetic biomarkers. Redox Biol. (2014) 2:206–10. 10.1016/j.redox.2013.12.026 24494194 PMC3909784

[78] Fossati G Moulding DA Spiller DG Moots RJ White MR Edwards SW. The mitochondrial network of human neutrophils: role in chemotaxis, phagocytosis, respiratory burst activation, and commitment to apoptosis. J Immunol. (2003) 170:1964–72. 10.4049/jimmunol.170.4.1964 12574365

[79] Patel S Fu S Mastio J Dominguez GA Purohit A Kossenkov A. Unique pattern of neutrophil migration and function during tumor progression. Nat Immunol. (2018) 19:1236–47. 10.1038/s41590-018-0229-5 30323345 PMC6195445

[80] Veglia F Tyurin VA Blasi M De Leo A Kossenkov AV Donthireddy L. Fatty acid transport protein 2 reprograms neutrophils in cancer. Nature. (2019) 569:73–8. 10.1038/s41586-019-1118-2 30996346 PMC6557120

[81] Lehuédé C Dupuy F Rabinovitch R Jones RG Siegel PM. Metabolic plasticity as a determinant of tumor growth and metastasis. Cancer Res. (2016) 76:5201–8. 10.1158/0008-5472.CAN-16-0266 27587539

[82] Rice CM Davies LC Subleski JJ Maio N Gonzalez-Cotto M Andrews C. Tumour-elicited neutrophils engage mitochondrial metabolism to circumvent nutrient limitations and maintain immune suppression. Nat Commun. (2018) 9:5099. 10.1038/s41467-018-07505-2 30504842 PMC6269473

[83] Gentles AJ Newman AM Liu CL Bratman SV Feng W Kim D. The prognostic landscape of genes and infiltrating immune cells across human cancers. Nat Med. (2015) 21:938–45. 10.1038/nm.3909 26193342 PMC4852857

[84] Ali HR Chlon L Pharoah PDP Markowetz F Caldas C. Patterns of immune infiltration in breast cancer and their clinical implications: a gene-expression-based retrospective study. PLoS Med. (2016) 13:e1002194. 10.1371/journal.pmed.1002194 27959923 PMC5154505

[85] Schalper KA Carleton M Zhou M Chen T Feng Y Huang SP. Elevated serum interleukin-8 is associated with enhanced intratumor neutrophils and reduced clinical benefit of immune-checkpoint inhibitors. Nat Med. (2020) 26:688–92. 10.1038/s41591-020-0856-x 32405062 PMC8127102

[86] Wang S Zhang Z Fang F Gao X Sun W Liu H. The neutrophil/lymphocyte ratio is an independent prognostic indicator in patients with bone metastasis. Oncol Lett. (2011) 2:735–40. 10.3892/ol.2011.304 22848258 PMC3406337

[87] Luo Y She DL Xiong H Fu SJ Yang L. Pretreatment neutrophil to lymphocyte ratio as a prognostic predictor of urologic tumors: a systematic review and meta-analysis. Medicine. (2015) 94:e1670. 10.1097/MD.0000000000001670 26448011 PMC4616750

[88] van Soest RJ Templeton AJ Vera-Badillo FE Mercier F Sonpavde G Amir E. Neutrophil-to-lymphocyte ratio as a prognostic biomarker for men with metastatic castration-resistant prostate cancer receiving first-line chemotherapy: data from two randomized phase III trials. Ann Oncol. (2015) 26:743–9. 10.1093/annonc/mdu569 25515657

[89] Walsh SR Cook EJ Goulder F Justin TA Keeling NJ. Neutrophil-lymphocyte ratio as a prognostic factor in colorectal cancer. J Surg Oncol. (2005) 91:181–4. 10.1002/jso.20329 16118772

[90] Ethier JL Desautels D Templeton A Shah PS Amir E. Prognostic role of neutrophil-to-lymphocyte ratio in breast cancer: a systematic review and meta-analysis. Breast Cancer Res. (2017) 19:2. 10.1186/s13058-016-0794-1 28057046 PMC5217326

[91] Zhou Q Hong L Zuo MZ He Z. Prognostic significance of neutrophil to lymphocyte ratio in ovarian cancer: evidence from 4,910 patients. Oncotarget. (2017) 8:68938–49. 10.18632/oncotarget.20196 28978169 PMC5620309

[92] Sharaiha RZ Halazun KJ Mirza F Port JL Lee PC Neugut AI. Elevated preoperative neutrophil:lymphocyte ratio as a predictor of postoperative disease recurrence in esophageal cancer. Ann Surg Oncol. (2011) 18:3362–9. 10.1245/s10434-011-1754-8 21547702 PMC3192937

[93] Huang QT Man QQ Hu J Yang YL Zhang YM Wang W. Prognostic significance of neutrophil-to-lymphocyte ratio in cervical cancer: a systematic review and meta-analysis of observational studies. Oncotarget. (2017) 8:16755–64. 10.18632/oncotarget.15157 28187430 PMC5369999

[94] Zhou Y Wei Q Fan J Cheng S Ding W Hua Z Prognostic role of the neutrophil-to-lymphocyte ratio in pancreatic cancer: a meta-analysis containing 8,252 patients. Clin Chim Acta. (2018) 479:181–9. 10.1016/j.cca.2018.01.024 29407690

[95] Yin X Xiao Y Li F Qi S Yin Z Gao J. Prognostic role of neutrophil-to-lymphocyte ratio in prostate cancer: a systematic review and meta-analysis. Medicine. (2016) 95:e2544. 10.1097/MD.0000000000002544 26817900 PMC4998274

[96] Vartolomei MD Porav-Hodade D Ferro M Mathieu R Abufaraj M Foerster B. Prognostic role of pretreatment neutrophil-to-lymphocyte ratio (NLR) in patients with non-muscle-invasive bladder cancer (NMIBC): a systematic review and meta-analysis. Urol Oncol. (2018) 36:389–99. 10.1016/j.urolonc.2018.05.014 29884342

[97] Gerratana L Basile D Toffoletto B Bulfoni M Zago S Magini A. Biologically driven cut-off definition of lymphocyte ratios in metastatic breast cancer and association with exosomal subpopulations and prognosis. Sci Rep. (2020) 10:7010. 10.1038/s41598-020-63291-2 32332763 PMC7181663

[98] Moldoveanu D Pravongviengkham V Best G Martinez C Hijal T Meguerditchian AN. Dynamic neutrophil-to-lymphocyte ratio: a novel prognosis measure for triple-negative breast cancer. Ann Surg Oncol. (2020). 10.1245/s10434-020-08302-2. [Epub ahead of print]. 32314154

[99] Marini O Spina C Mimiola E Cassaro A Malerba G Todeschini G. Identification of granulocytic myeloid-derived suppressor cells (G-MDSCs) in the peripheral blood of hodgkin and non-hodgkin lymphoma patients. Oncotarget. (2016) 7:27676–88. 10.18632/oncotarget.8507 27050283 PMC5053680

[100] Brandau S Trellakis S Bruderek K Schmaltz D Steller G Elian M. Myeloid-derived suppressor cells in the peripheral blood of cancer patients contain a subset of immature neutrophils with impaired migratory properties. J Leukoc Biol. (2011) 89:311–7. 10.1189/jlb.0310162 21106641

[101] Liu Y Hu Y Gu F Liang J Zeng Y Hong X. Phenotypic and clinical characterization of low density neutrophils in patients with advanced lung adenocarcinoma. Oncotarget. (2017) 8:90969–78. 10.18632/oncotarget.18771 29207617 PMC5710898

[102] Shaul ME Eyal O Guglietta S Aloni P Zlotnik A Forkosh E. Circulating neutrophil subsets in advanced lung cancer patients exhibit unique immune signature and relate to prognosis. FASEB J. (2020) 34:4204–18. 10.1096/fj.201902467R 31957112

[103] Millrud CR Kagedal A Kumlien Georen S Winqvist O Uddman R Razavi R. NET-producing CD16 (high) CD62L(dim) neutrophils migrate to tumor sites and predict improved survival in patients with HNSCC. Int J Cancer. (2017) 140:2557–67. 10.1002/ijc.30671 28247912

