# Supplementary material for: Neutrophils: Orchestrators of the Malignant Phenotype
Source: Front Immunol. 2020 Aug 11;11:1778. doi: 10.3389/fimmu.2020.01778 (PMC7433712; doi:10.3389/fimmu.2020.01778)
Supplement: Supplementary file 1 [file Table_1.pdf]

Table 1: Distinct Neutrophil Subsets and their Associated Functions

Neutrophil Subset	Function	Maturation Stage	Cancer Model	Reference
HDN	Anti-tumor<br>H_2O_2 dependant cytotoxicity against TRPM2 expressing tumor cells	Mature	Murine breast cancer (AT3, 4T1)<br>Murine lung cancer (LLC)<br>Human breast cancer (MDA-231)	Gershkovitz *et al.*, 2018a
HDN	Anti-tumor<br>Mesenchymal cancer cells are highly susceptible to neutrophil-mediated killing	Mature	Murine breast cancer (4T1)	Gershkovitz *et al.*, 2018b
HDN	Anti-tumor<br>H_2O_2 cytotoxicity against RAGE-expressing cancer cells	Mature	Murine breast cancer (AT3, E0771)<br>Murine lung cancer (LLC)<br>Murine melanoma (B16-F10)	Sionov *et al.*, 2019
LDN	Pro-tumor<br>T cell suppression	Mixture of Mature and immature neutrophils	Murine breast cancer (4T1)	Sagiv *et al.*, 2019
LDN	Pro-tumor<br>Increased NETosis, N2 neutrophil polarization	Unknown	Spontaneous intestinal tumor model<br>C57BL6/J – ApcMin/J	Guglietta *et al.*, 2015
LDN	Pro-metastatic<br>Increased liver metastasis, enhanced metabolic flexibility, enhanced NETosis	Predominately immature	Murine breast cancer<br>(liver-metastatic variant – 4T1)<br>Colorectal cancer (CT26)	Hsu *et al.*, 2015
